# Why Consumers Prefer Green Friariello Pepper: Changes in the Protein and Metabolite Profiles Along the Ripening

**DOI:** 10.3389/fpls.2021.668562

**Published:** 2021-04-30

**Authors:** Maria Tartaglia, Rosaria Sciarrillo, Daniela Zuzolo, Angela Amoresano, Anna Illiano, Gabriella Pinto, Jesús V. Jorrín-Novo, Carmine Guarino

**Affiliations:** ^1^Department of Science and Technology, University of Sannio, Benevento, Italy; ^2^Department of Chemical Sciences, University of Naples Federico II, Naples, Italy; ^3^CEINGE Advanced Biotechnologies, University of Naples Federico II, Naples, Italy; ^4^Agroforestry and Plant Biochemistry, Proteomics and Systems Biology, Department of Biochemistry and Molecular Biology, University of Córdoba, UCO-CeiA3, Córdoba, Spain

**Keywords:** proteomics, metabolomics, fruit ripening, agro-biodiversity, Friariello pepper, *Capsicum annuum* L.

## Abstract

Fruit ripening is a physiologically complex process altering texture, color, flavor, nutritional value, and aroma. However, some fruits are consumed at an early stage of ripening due to the very peculiar characteristics varying during ripening. An example is a particular ecotype of pepper, the Friariello pepper, among the most important representatives of Campania (Southern Italy) agro-alimentary culture. In this study, for the first time, the physiological variations during Friariello ripening (green, veraison, and fully ripe) were evaluated by hyphenated mass spectrometric techniques in a proteomic and metabolomic approach. We found that Lutein and Thaumatin are particularly abundant in the green Friariello. Friariello at an early stage of ripening, is rich in volatile compounds like butanol, 1 3 5-cycloheptatriene, dimethylheptane, α-pinene, furan-2-penthyl, ethylhexanol, 3-carene, detected by gas chromatography-mass spectrometry (GC-MS) analysis, which give it the peculiar fresh and pleasant taste. The detected features of Friariello may justify its preferential consumption in the early ripening stage and outline new knowledge aimed at preserving specific agro-cultural heritage.

## Introduction

Italy claims a very high biodiversity in plant species and crops due to diversified biogeographics and climatic conditions which reflect also in agrodiversity, where the millenary rural culture and mixture of ethnicity, in their long history, have given rise to several agrifood productions. This wide food variety sink from agriculture means an enormous source for healthy alimentation and well-being, which also include cultural identities and contributes to make cultural landscapes (Kahane et al., 2013).

The pepper (*Capsicum annuum* L.), imported from tropical regions, is nowadays one of the most widespread and appreciated species for their economical, nutritional value and for ethnobotanical uses (Liu et al., 2019; Prigioniero et al., 2020). In fact peppers are an excellent source of essential compounds for human nutrition such as vitamin C, B, antioxidants, carotenoids and minerals, mainly potassium, iron, magnesium and calcium. Furthermore, the high content of water and fibers and the low lipid content make them advisable for low-calorie diets (Deepa et al., 2007; Martí et al., 2011; Emmanuel-Ikpeme, 2014). Several varieties of peppers, with peculiar organoleptic characteristics and high commercial value, and niche ecotypes closely linked to local cultural and ethnobotanical traditions, are grown in Italy. Ripening is the process that makes fruits desirable for consumption through the change in the composition and color, the cell walls degradation and the consequent softening of the pulp, the conversion of the starch into sugar, the production of volatile compounds that improve smell and flavor (Osorio et al., 2012; Chaki et al., 2015). Those are all physiological processes well regulated by the plant. The molecular, proteomic and metabolomic pathways involved in fruit ripening vary between species; while the climacteric fruit ripening process, has been extensively studied; ripening of non-climacteric fruit, like pepper, is poorly understood. Transcriptome analysis and metabolite profiling showed a tight regulation during fruit maturation but a more direct correlation is expected for proteins and metabolites (Palma et al., 2011).

Friariello is a pepper ecotype particularly widespread and appreciated in Campania, Southern Italy, for its organoleptic characteristics, strong and sweet flavor and high digestibility (Fratianni et al., 2020). The fruit is a berry of commercial size between 6 and 10 cm in length characterized by sweet taste and intense aroma. Pepper, a non-climacteric fruit, does not ripen under the ethylene input, Friariello is usually harvested and consumed at an early maturation stage when the fruit has reached its maximum size but is still green, however, the consumer demand is recently shifting on complete ripening stage fruits, harvested when the conversion from chloroplasts to cromoplasts has been completed and the yellow, orange or red pepper has reached maximum levels of pigments, vitamins and phenolic compounds (Serrano et al., 1995). Friariello berries, conical and elongated, can have a variable number of lobes: three in the “Nocerese” type, two in the “Torrese” or “Napoletana” type and only one in the “Sigaretta” type. Although some seed companies have placed on the market Friariello hybrids with resistance to some biotic stresses, most of the crops are propagated with seeds deriving from self-production, allowing the almost unaltered conservation of historical germoplasms well adapted to the territory. There are no data in the literature on proteomic and metabolomic variations during the ripening process of this variety so little widespread, however, with the object of the biodiversity conservation and the food-traditional and territorial cultivations preservation, it is appropriate and interesting to characterize this fruit not only at an agronomic level. In this work, the “Sigaretta” cultivar has been selected and investigated. The total protein expression and the metabolites quantitative variation in the green consumption stage, in the veraison stage, and in the complete ripening stage of the pepper have been evaluated. These analyzes were carried out to evaluate the physiological changes in color, texture, flavor and aroma in ripening Friariello and to understand why it is preferentially consumed in the immature stage.

## Materials and Methods

### Fruit Material

The pepper fruits were harvested in open field in a typical area of cultivation located in the Agro Nocerino-Sarnese (Lon 14°33′38.22″ E; Lat 40°43′38.22″N). In this area have been cultivated a small number of selected local ecotypes. The climate in this area is typically Mediterranean, the coldest month is January (8°C medium temperature) and the warmest is August (23.8°C), the medium annual rainfall is about 900 mm mostly distributed in the autumn/winter months. The cultivation field is characterized by soil deep, well drained, sandy loam, weakly alkaline. Seeds for cultivation were originated in the same farm from previous year crop. The cultivation start from seeds in greenhouse with a daily temperature ranging between 18 and 27°C and an average relative humidity of 85%, and the plantlets were transplanted in open field after 40 days from emergence in number of 20.000 plants per hectare. The cultivation in open field started in April (2020) and three fruits, from 10 plants chosen at random, were hand-collected at 90 (commercial maturity – green fruit G), 120 (reddening phase-veraison G/R) and 150 days (physiological maturity – full red R) after in field transplant. During the growing season is provided water by drip irrigation and fertilized with nitrogen and potassium (phosphorus is provided preplant). Fertilization during open field growth was carried out with a binary fertilizer NK (15.0.30) in a single administration after transplantation.

### Proteomics Analysis Methods

#### Protein Extraction

The sampled fruits were washed in deionized water, dried, cleaned of seeds and petiole, and finely pulverized in liquid nitrogen. The extraction of total proteins was carried out by using a modified version of classic tricarboxylic acid (TCA)-Acetone extraction with the addition of 1% of polyvinylpyrrolidone from pulverized material (0.2 g) of the three samples G, G/R and R (Wang et al., 2003). After the precipitation in ammonium acetate in methanol and three acetone washes, the pellet was quickly dried under vacuum. The protein pellet obtained, was then solubilized in a solubilization buffer (7M urea, 2M thiourea, 4% (w/v) CHAPS, 40 mM DTT, and 0.5% IPG buffer) for 1 h at room temperature and centrifuged to eliminate the insoluble material (12,000 × *g* for 5 min at 4°C). The protein concentration was estimated by Bradford method with bovine serum albumin as a standard.

#### Sodium Dodecyl Sulphate–PolyAcrylamide Gel Electrophoresis (SDS–PAGE)

Protein extracts from pepper samples were kept in hot water (90°C) in SDS loading buffer and loaded onto 12% SDS–PAGE gel in agreement to Laemmli et al. (Laemmli, 1970). Gels were run for 30 min at 60-V and around 2 h at 130-V at room temperature. The buffer used for the electrophoretic run was a 1× Tris/Tricine/SDS Running Buffer (Bio-Rad). The gel was stained with Coomassie Stain (EzBlueRstain reagent Sigma-Aldrich) and then washed with distilled-water and destaining solution (methanol 30% and acetic acid 10% in water) overnight. For each sample (green, green/red, and red), three biological replicates were loaded on three separate gels.

#### *In situ* Digestion

SDS-PAGE bands (horizontal slices) were excised from the gel lane and destained by using three consecutive cycles of 0.1 M NH_4_HCO_3_ at pH 8.0 and acetonitrile (ACN), followed by reduction (10 mM DTT in 100 mM NH_4_HCO_3_, 45 min, and 56 °C) and alkylation (55 mM IAM in 100 mM NH_4_HCO_3_, 30 min, and RT). The gel pieces were washed with three further cycles of 100 mM NH_4_HCO_3_ of pH 8.0 and ACN. Finally, the gel pieces were subjected to the enzymatic hydrolysis by covering them with 40 μL sequencing grade modified trypsin (10 ng μL^–1^ trypsin; 10 mM NH_4_HCO_3_) and incubated overnight at 37 °C. Peptide mixtures were eluted, vacuum-dried, and resuspended in 2% ACN acidified with 0.1% formic acid (HCOOH) previous to the LC-MS/MS (liquid chromatography-mass spectrometry and liquid chromatography-tandem mass spectrometry) analysis.

#### LC-MS/MS Analysis

Peptide mixtures were analyzed by a 6520 Accurate-Mass Q-TOF LC/MS system (Agilent Technologies) equipped with a 1200 HPLC system and a chip cube (Agilent Technologies). After loading, the peptide mixture (1 μL) was concentrated and desalted at flow rate of 4 μL/min in a 40 nL enrichment column with 0.1% HCOOH as eluent. The sample was then fractionated on a C18 reverse phase capillary column (75 μm × 43 mm in the Agilent Technologies chip) at flow rate of 400 nL/min, with a linear gradient of eluent B (0.1% HCOOH in 95% ACN) in A (0.1% HCOOH in 2% ACN) from 5 to 80% in 50 min. Peptide analysis was performed using data-dependent acquisition of one MS scan (mass range m/z 300–2,400) followed by MS/MS scan of the five most abundant ions in each MS scan. MS/MS spectra were measured automatically when the MS signal was greater than the threshold of 50,000 counts. Double, triple and quadruple charge ions were preferably isolated and fragmented over singly charged ions. Data were acquired through Mass Hunter software (Agilent Technologies). The acquired data, containing MS and MS/MS spectra, were transformed in.mgf format and used for protein identification with a licensed version of Mascot Software^1^. Mascot search parameters included: *C. annuum* as database (Capsicum_UP000222542); trypsin as enzyme, allowed number of missed cleavage 3; carbamidomethyl, C as fixed modifications; oxidation of methionine (oxidation of methionine, Gln pyro-Glu (N-term Q) as variable modifications; 10 ppm MS tolerance, 0.6 Da MS/MS tolerance and peptide charge, from +2 to +4. Every protein was selected as significant when at least 2 peptides displayed a *p* value < 0.05. The relative quantification was performed in agreement to the literature data (Ishihama et al., 2005).

#### Principal Component Analysis

A two-dimensional biplot showing associations between experimental samples and protein spots were generated by principal component analysis (PCA) in R environment (R Core Team, 2018)^2^ to depict the proteins pattern. The biplot is a 2D graphical representation used with multivariate data sets to describe and display the relationships between observations and variables which are expressed as dots and rays, respectively. Rays represent the variables (proteins) and the lengths of the rays are directly proportional to the variance of the corresponding protein included in the two components displayed. The distance between two vectors depicts the link (vector connecting the endpoints) between them (Zuzolo et al., 2020). To deal with missing values (which can heavily disturb multivariate statistics) we only considered “consistent spots,” which were those present in the three sample type (Meunier et al., 2007; Valledor and Jorrín, 2011). Samples and protein spots were plotted in the first two component spaces (PC1 and PC2).

### Metabolomics Analysis Methods

#### Solid-Liquid Phase Extraction Coupled to MS Analysis

The metabolites of pepper along the aging phases were extracted by a solid-liquid phase extraction (SLE) and analyzed by mass spectrometry (MS). The whole pepper at different aging level (G; G/R; R) was ground with a mortar under liquid nitrogen. Each sample (1 g) was extracted twice in an Ultra Turrax homogeniser with acetone/hexane (1/1). The pellet was discarded while the supernatant (a total volume of 40 mL) was dried by a continuous stream of nitrogen and resuspended in volume of 1 ml of n-hexane and acetone (1:1). Three aliquots of hexane/acetone extract were analyzed by matrix-assisted laser desorption/ionization (MALDI)-TOF/TOF, LC-TOF, and gas chromatography-mass spectrometry (GC-MS) analysis.

#### Matrix-Assisted Laser Desorption/Ionization–Mass Spectrometry Analysis

Matrix-assisted laser desorption/ionization–MS analysis was performed on a 5,800 MALDI-TOF-TOF equipped with a nitrogen laser (337 nm) (AB SCIEX, Milan, Italy). Aliquots of extract (1 μL) were mixed (1:1, v/v) with a solution of 2.5 dihydroxybenzoic acid (DHB, Sigma-Aldrich, Milan, Italy) at a concentration of 20 mg mL^–1^ in ACN:water (50:50) solution. The MS spectra were acquired in reflector positive ion mode, by using a mass (m/z) range of 100–4,000 Da. Laser power was set to 4,000 V for MS spectra acquisition and maintained fixed for all the analyses. Each spectrum represented the sum of 10,000 laser pulses from randomly chosen spots per sample position. For CID experiments, ambient air was used as collision gas with medium pressure of 10–6 Torr. The data were reported as monoisotopic masses.

#### LC-MS Analysis

LC-MS analysis was performed by an Agilent HPLC system (1260 Series) on a reverse-phase C18 column (Agilent Life Sciences Extend-C18, 2.1 × 50 mm, 1.8 μm) coupled to an Agilent 6230 Time-of-flight (TOF) mass spectrometer. The HPLC separation was carried out by using water and ACN as mobile phases A and B, respectively, both acidified with 0.1% HCOOH. The injection volume was 20 μL. A linear gradient was applied over 42 min (0–2 min: 2% B, 2–8 min: 2–40% B, 8–20 min: 40%–60% B, 20–30 min: 60–80%, 30–40 min, 80–95%) at a flow rate of 0.3 mL min^–1^. The MS source was an electrospray ionization (ESI) interface in the positive ion mode with capillary voltage of 3,000 V, gas temperature at 325°C, dry gas (N_2_) flow at 5 L min^–1^ and the nebulizer pressure at 35 psi. The MS spectra were acquired in a mass range of 150–1,000 m/z with a rate of 1 spectrum/s, time of 1,000 ms/spectrum and 9,961 transient/spectrum.

#### Analysis of Volatile and Semi-Volatile Compounds (Solid Phase Micro-Extraction or Solid-Liquid Extraction/GC-MS)

*Volatile component* was extracted by solid phase micro-extraction (SPME) protocol toward the use of a 2 cm fiber (df 50/30 μm) supported by divinylbenzene/carboxen/polydimethylsiloxane (DVB/CAR/PDMS) (Supelco and Sigma-Aldrich). An aliquot of pepper (1 g) opportunely ground under liquid nitrogen was exposited to the fiber for 40 min at 60°C. The fiber was then exposed in GC injector at a temperature of 230°C for 3 min (desorption).

*The semi-volatile component* was obtained as reported below. An aliquot of 1 μL was injected into the GC-instrument.

All the GC analyses were performed using Agilent GC 6890, coupled with a 5973 MS detector. The column used was an HP-5 capillary (30 m × 0.25 mm, 0.25 mM, 5% polisilarilene 95% PDMS). Helium was used as carrier gas, at a rate of 1.0 mL min^–1^. The GC injector was maintained at 230°C, while the oven temperature was held at 60°C for 3 min and then increased to 150°C at 10 °C/min, increasing to 230°C at 14°C/min and finally to 280°C at 15°C/min held for 5 min for a total separation time of 23 min. The analyzer temperature was kept at 250°C. The collision energy was set to a value of 70 eV and fragment ions generated were analyzed mass range 20–450 m/z.

The identification of each compound was based on the combination of retention time and fragmentation spectra matching those collected into NIST 05 Mass Spectral Library. The identification was reliable when the matching values were higher than 700 according to the NIST guide lines (Stein, 1994).

## Results

### Experimental Workflow

**FIGURE 1:**
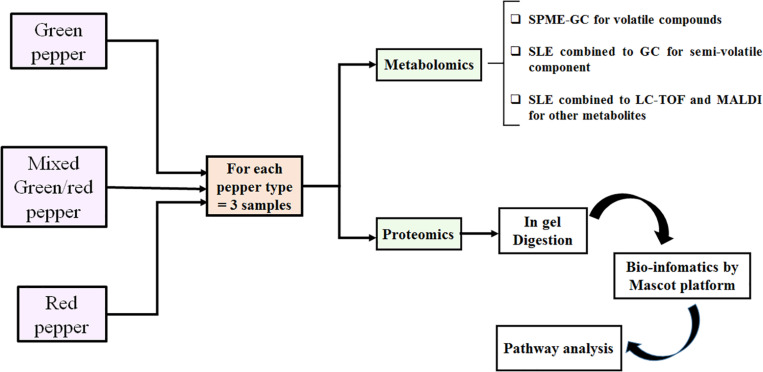
Workflow of experimental strategy followed for metabolomics and proteomics analyses.

Molecular analysis of postharvest processes and aging of sp. *C. annuum* was followed by using both proteomics and metabolomics approaches at different stages (Figure 1). For proteomics, an *in-situ* digestion of SDS fractionated protein extracts was performed for each sample (green, mixed green/red, and red peppers) to monitor the differential proteins abundance along all the time of aging. For metabolomics, the metabolites were extracted by SLE and the supernatants were analyzed by MALDI-MS, LC-MS, and GC. The volatile component was investigated by coupling SPME and GC-MS.

### Proteomic Profiles of Friariello Pepper at Different Maturation Stages

**FIGURE 2:**
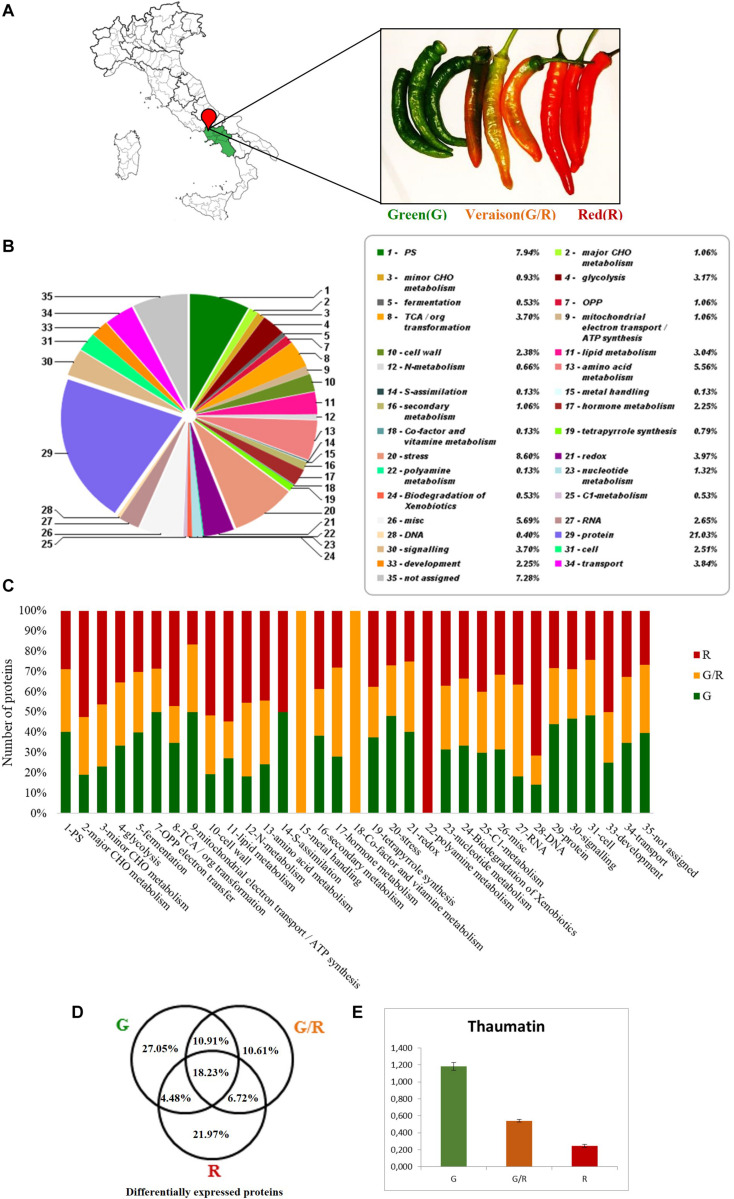
**(A)** Friarielli “Sigaretta” variety collected in the three stages of ripening: green (G), green–red (G/R), ripe (R), used for the analyzes. **(B)** Proteins differentially expressed by percentage abundance of functional classes by Mercator4 V2.0 (https://mapman.gabipd.org/app/mercator). **(C)** Representative histogram of abundances by functional classes in the three stages of maturation. **(D)** Venn diagrams which represents the univocal or shared expression of the total of differentially expressed proteins. **(E)** Expression of Thaumatin protein (A0A1U8G8A9) in Friariello green–green/red–red samples.

SDS-PAGE bands for each lane of pepper were *in situ* digested for a large-scale proteomics study by a label free differential quantification (Figure 2A). Proteomic analysis allowed the identification of 669 proteins (Supplementary Table 1) in the G, G/R, and R samples. These proteins have been divided using Mercator4 V2.0^3^ into functional classes and represented in Figures 2B,C. Among these proteins, 27.05% is present exclusively in the G stage, 10.61% in the G/R stage and 21.97% in the R one. 10.91% of proteins are shared in the G–G/R stages, 6.72% in the G/R-R stages and 4.48% in the G-R stages. 21.97% of differentially expressed proteins are shared in all ripening stages (Figure 2D).

### Principal Component Analysis

**FIGURE 3:**
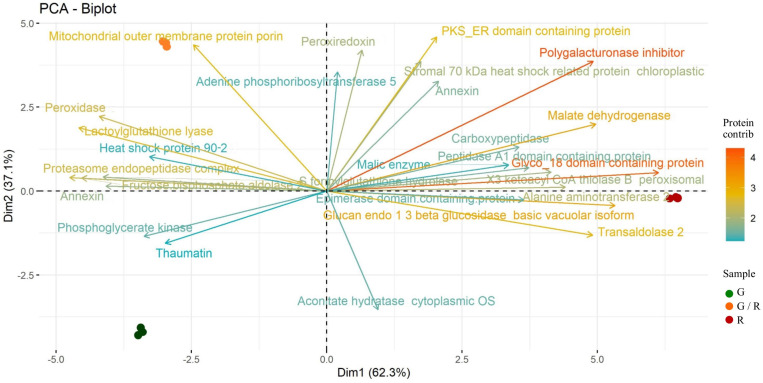
Two-dimensional principal component analysis (PCA) biplot showing associations between experimental samples and protein abundance generated by PCA. The 1st and 2nd component (PC 1 and PC2, respectively) are displayed. Samples are distributed according to their ripening stage: G (green), G/R (green/red), and R (red). The 30 proteins (vectors) with highest contribution are individually labeled.

The PCA results are very clear, with homogeneous replicates. In addition, the employment of the PCA biplot showed the separation of samples into their ripening stages. The PCA biplot (Figure 3) depicts the association between the protein patterns and samples and it explains almost all variability (99.4%). The 30 proteins (vectors) with highest contribution are individually labeled. The first PC accounts for 62.3% of total variability and displays a separation between the most ripened sweet pepper (R) and the others. The protein expression pattern of R is visible as vectors which tend to cluster together. Among this group of proteins, Polygalacturonase inhibitor, Glucan endo-1,3-beta-glucosidase, basic vacuolar isoform, Malate dehydrogenase and Glyco_18 domain-containing protein have the highest scores on the first principal component. On the other hand, an antithetic group of proteins mainly expressed as Peroxidase and Lactoylglutathione lyase is also distinguishable. The variances explained by PC2 is 37.1% and it clearly separates G and G/R. The protein profile of G/R is mainly featured by Mitochondrial outer membrane protein porin and peroxidase, whereas the G protein profile is dominated by Thaumatin and Phosphoglycerate kinase.

### Metebolic Profiles of Friariello Pepper at Different Maturation Stages

The hexane/acetone extracts were loaded on MALDI plate and crystallized with DHB matrix as reported in Materials and Methods section. MALDI spectra of G, G/R and R peppers were recorded as an average of five replicates at 4,000 pulsed laser and 10,000 shots of accumulation and reported in a 400–1,300 mass range (Supplementary Figure 1). The metabolite identification was reported in Table 1 where a X was assigned to species detected into each sample. The confirmation of assignment was carried out by MALDI-TOF/TOF analysis and the fragment ions were reported for each fragmented analyte (Table 1). The MS/MS analysis allowed to underline that the most abundant peaks belonged to the following classes: carotenoids/saponins, chlorophylls and lipids differently abundant in three samples (Table 1).

**TABLE 1:** List of the theoretical and experimental masses of parent ions MH^+^ m/z or M^⋅+^ m/z is presented.

Carotenoids and saponins	MW Da	MH+ m/z	M^⋅+^	Green	Green/red	Red	Fragment ions in ms/ms spectra (m/z)
Capsorubin-H_2_O	582.4	583.4		x	x	x	429.5;446;491.8;564.2
Soyasaponin III	796.5	797.5		x	x	x	635.4; 519.6; 357;3
Soyasaponin V	958.6	959.6		x	x	x	797.8; 681.6; 519.5; 405.3
Steroidal Saponins	939.5	940.5		x	x	–	
Steroidal Saponins	1,034.7	1,035.7		x	x	–	
Steroidal Saponins+sodium adduct	1,056.7	1,057.7		x	x	x	457.3;599.5;779.6;775.5;901.8
Steroidal Saponins	1,070.7	1,071.7		x	x	x	39;319.3;599.6,791.6
Derived carotenoid	482	483		x	–	x	193.1;329.1;345.17;346.17;439.2
Stheroidal saponins		937.6		x	x	x	91;184;283,3;311;347;405;659.5; 681.4;775.6
**Chlorophylls**							
Pheophorbide A	592.7	593.4		x	x	x	459.5;519.5;533.6
Pheophorbide B	606.7	607.7		x	x	–	
Pheophtyn A	871.2	–	871.6	x	x	x	460;533;593;839;869
Pheophtyn B	885.2	–	885.6	x	x	–	
Chlorophyll A	892.5	–	893.5	x	x	x	
Chlorophyll B	906.5	–	907.5	x	–	–	
Chlorophyll D	895.5	896.5		–	–	–	
**Lipids**							
WE(22:6/18:3)	574.5	575.5		x	x	x	43;55;57;67,69;71;83;85;95;97; 99.2;109.2;123,2;137,2; 239,3;263.3;417;552;553.1;
WE(22:6/20:5)	598.4	599.4		x	x	x	67;81;95.2;109.2;123.2; 221.3;263.4;391.5;507.7
DG(16:1/20:3)	616.5	617.4		–	–	x	
DG(18:3/20:5)	636.4	637.4		x	x	x	
DG(18:2)/20:5)	638.4	639.4		x	x	x	
PE(18:3/18:3)	735.6	736.6		x	x	x	
PE(18:2/18:3)	737.6	738.6		x	x	x	
PC(16:0/18:2	757.6	758.6		–	x	x	
PC(18:2/18:3)	779.6	780.6		–	x	x	86;147,1;184,2;597,7;721,7
PC(18:2/18:2)	781.6	782.6		–	x	x	
PC(22:2/18:2)	837.6	838.5		x	x	x	473.4;487.4;516.4;533.4;537.560.4; 561.5;595.3;811.8;813.7
PC(20:5/22:6)	851.5	852.5		–	x	–	
TG(18:2/20:5/16:0)	876.6	877,7		x	x	x	39;576.2;597;621.8;831;875
TG(18:2/18:2/20:5)	900.7	901.7		x	x	x	39;600;622;899.2
TG(22:0/22:6/22:6) sodium adduct +Ox	1,072.7	1,073.7		x	–	–	
Pyrophosphate SM C24H50N2O13P2Na	658.3	659.3		x	x	x	109.2;184.2;262.4;335.3;505.2;551; 583.6;599.6;639.3
SM(d18:2/24:0)	812.5	813.5		x	–	x	39;184.2;462.4;533.4;535.5;

*For each analyte the peak area is reported.*

Indeed, Pheophtyn A (Mg-free Chlorophyll A) at 871.5 m/z was the most abundant species with 100% relative abundance in the green and mixed samples whereas the dehydrated form of capsorubin (583.4 m/z) was that for red pepper. It was observed that Chlorophylls A, B derived analytes (e.g., Pheophytin and pheoporbide) were abundant especially in green peppers while carotenoids and saponins increased in R ones thus suggesting a different relevance of these analytes along the maturation in agreement with proteomics results. Phosphatidylcholine (PC) and phosphatidylethanolamine (PE) were also detected in all MALDI spectra as well as diacylglycerol, triacylglycerol and cuticle wax esters (575.5 m/z and 599.4). For all of them the most abundant peaks were interestingly recorded in green/red peppers suggesting a more intense synthesis of lipids or a slower degradation of such molecules during the intermediate stage (Table 1).

The low ionization efficiency of carotenoids by MALDI ionization suggested the need of a further analysis of hexane/acetone extracts by LC-MS. Actually, the hexane extracts were also injected into a LC-TOF apparatus and the peak areas recorded along the time of linear gradient as shown in Table 2.

**TABLE 2:** List of the most abundant metabolites identified by LC-MS analysis.

RT (min)	m/z	Small molecule	Green	Green/red	Red
28.2/28.5	270.3	Capsi-amide	–	–	1,440,013.88
22.1	295.2	Nordihydrocapsiate	1,59,038.5	6,63,820.19	2,38,754.39
22.1	277.2	Nordihydrocapsiate – H_2_O	52,936.67	2,23,425.33	77,726.54
20.5	319.2	Homocapsaicin	–	12,828.48	–
26.6	468.4	Capsaicin beta-D-glucopyranoside	–	–	6,812.3
**Carotenoids**					
27.8	601.4	Capsorubin	–		10,822.42
28.2/28.5	585.4	Capsanthin	–	2,99,327.5	6,97,687.25
30.2	583.4	Capsorubin-H_2_O	–	4,003.27	1,42,768.22
28.2/28.5	584.4	Violaxanthin – 16	–	77,868.1	2,10,134.52
30.2	584.4	Neoxanthin – 16	26,784.04	77,868.13	2,10,045.31
30.2	600.4	Neoxanthin	13,015.36	11,133.48	40,664.27
26.6	568.5	Zeaxanthin	1,05,436.52	1,47,598.77	1,39,533.89
31.3	568.4	Lutein	4,71,702.97	1,55,099.55	1,55,099.55
31.3	551.4	Lutein – H_2_O	2,78,377.66	–	–

The most abundant signals were related to carotenoids and other small molecules characterizing the *Capsicum* sp. Capsi-amide and Capsaicin beta-D-Glucopyranoside were exclusively detected in green peppers whereas homocapsaicin in green/red peppers. Nordihydrocapsiate and its dehydrated form were shared along the samples with the higher abundance in green/red peppers (Table 2). On the other hand, all the carotenoids, e.g., capsorubin, capsanthin, violaxanthin, neoxanthin, zeaxanthin, and derived were exclusively detected in red/mixed peppers or they increased along the maturation (Table 2). An exception was observed for lutein and its dehydrated form that resulted to decrease along the maturation as reported by others (Gómez-García and Ochoa-Alejo, 2013).

**FIGURE 4:**
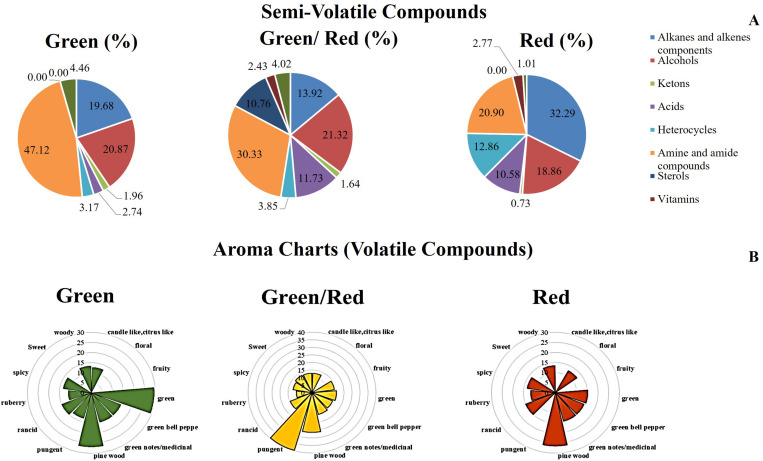
**(A)** Cake representation of distribution (%) of Semi-volatile classes detected in each sample (Supplementary Table 2). **(B)** Aroma chart depicting the sweet pepper (*Capsicum annuum* L.) aroma properties of green, green/red, and red development stage. Axis showing peak area is ln transformed (Supplementary Table 3).

An analysis of semi-volatile was carried out coupling the SLE extraction with GC-MS analysis. TIC chromatograms by GC-MS analysis of hexane extracts from G, G/R, and R peppers were overlapped (Supplementary Figure 2). The highest TIC was recorded for red peppers whereas the lowest was for mixed peppers. The most abundant classes of molecules e. g. alkanes and alkenes components, alcohols, ketons, etc., were grouped in Supplementary Table 2. A cake representation of percentages of the molecule classes was reported in Figure 4A. Almost 50% was represented by amine and amide compounds for green peppers with % decreasing along the maturation stages. The main classes were instead eterocycles for red peppers (Figure 4A). Moreover tocopherol was abundant in R and G/R peppers while it was absent in G peppers. The volatile component was extracted by SPME and desorbed by the fiber for 3 min in a GC-MS apparatus. TIC chromatograms by GC-MS analysis of volatile compounds extracted by SPME fiber from G, G/R, and R peppers were overlapped (Supplementary Figure 3). Some volatile molecules were described also by other as characterizing the green pepper such as isobutyl-3-methoxypyrazine (Mutarutwa et al., 2018) while other molecules, e.g., 3-hydroxy-1-propenyl-cyclopentanone were more abundant in R peppers giving the spicy aroma. The highest percentage was represented from the alcohol classis with a profile decreasing along the maturation stage as for alkans/alkenes and heterocycles/aromatic compounds. An increase of volatile component during the aging was observed for nitrogenate compounds and ketons/aldehydes whereas terpenes showed a constant trend (Figure 4B, Supplementary Table 3).

## Discussion

The pepper, consumed as a fresh vegetable, cooked or spice, is an excellent source of metabolites with beneficial effects on health, such as tocopherols (vitamin E), ascorbic acid (vitamin C), carotenoids (provitamin A), capsaicinoids, and flavonoids. However, diverse varieties of *Capsicum* have, in addition to distinctive morphological characters, several different metabolites at different concentrations (Wahyuni et al., 2013). In addition, during the fruit ripening process there is a complicated and cooperative regulation of primary and secondary metabolism which corresponds to variations in fruit color variations in fruit color, flavor, texture, and nutritional value.

### Friariello Ripening Process: Color and Texture Variation

The toning from green to red, that occurs during the ripening of *C. annuum* fruits, is due to the conversion from chloroplast to chromoplast in the exocarp (Berry et al., 2019). As expected, our results have shown that numerous proteins involved in the first functional group (PS) are significantly differentially abundant in the three stages of maturation (Supplementary Table 1). These are mainly involved with the chloroplast function, photosynthesis and oxygen evolution. In particular, Chlorophyll A-B binding protein, Oxygen-evolving enhancer protein, proteins associated with the photosystems I and II reaction centers, RuBisCO, Phosphoglyceric kinase, Fructose-bisphosphate aldolase, are abundant in G stage or show a marked down-regulation in G/R and especially in R stage. This down-regulation is an indication of an obvious reduced photosynthetic activity consistent with the data present in the literature (Du et al., 2016). During the transition from chloroplast to chromoplast, one of the most noticeable metabolic change is the loss of chlorophyll and the accumulation of carotenoids in our peppers as shown in Tables 1, 2 (Bian et al., 2011). The antioxidant system plays a crucial role in the ripening process, as seen in the literature (Jimenez et al., 2002). Hence, in accordance with our proteomics results (Supplementary Table 1), there is an up-regulation of antioxidant enzymes during the ripening process. In higher plants, carotenoids are synthesized in the plastid from isopentenyl pyrophosphate (IPP, C5) via the methylerythritol-4-phosphate pathway. Geranylgeranyl pyrophosphate is the lipid precursor of phytoene synthase. The phytoene then undergoes a series of dehydrogenations to produce lycopene which can be cyclized into α-carotene and β-carotene. In the carotenoid pathway in *Capsicum*, xanthophylls, zeaxanthin, violaxanthin are produced from β-carotene. The final products found in high concentrations in red peppers samples are capsorubin and capsanthin. To form capsanthin and capsorubin, the action of capsanthin/capsorubin synthase (CCS) is required (Berry et al., 2019). According to our proteomic and metabolomic outcomes, the CCS enzyme is expressed in the early Friariello ripening stage and its products accumulate during the advanced phases (Table 2). Zeaxanthin, but especially Lutein, are particularly abundant in the green Friariello. Lutein antioxidant beneficial effects on human health are well known, however, its biologically effective dose is difficult to achieve due to its low bioaccessibility and bioavailability in food sources (Ochoa Becerra et al., 2020). Given the accumulation of lutein in the green Friariello, the consumption of this fruit in an early stage of ripeness could have a nutraceutical power, positively reflecting on human health (Granado et al., 2003).

The change in fruit texture during ripening is mainly due to the cell wall main components depolymerization promoted by hydrolytic enzymes (Goulao and Oliveira, 2008). Our data have highlighted that different proteins, belonging to this functional class (pectinesterase, Pectin acetylesterase, L-ascorbate oxidase, xyloglucan endotransglucosylase, expansin, pectate lyase, beta-galactosidase, endoglucanase, and alpha-L-arabinofuranosidase) are present exclusively, or in any case up-regulated, in the fully ripe pepper sample. The beta-galactosidase enzyme is particularly abundant in the green/red and red stages. This removes non-reducing terminal β-D-galactosyl residues of hemicellulose and pectin from the cell wall by increasing the degree of porosity of the cell wall and facilitating the lytic action on the wall polysaccharides by other enzymes (Yang et al., 2018). A peculiar result is the increase of the Polygalacturonase inhibitor expression during the ripening of Friariello pepper. This protein inhibits the effect of polygalacturonase, enzymes that degrade the polygalacturonan component of plant cell walls. However, the inhibition effect is specific for polygalacturonases produced by pathogenic bacteria and fungi, but has no effect on endogenous plant polygalacturonases that naturally participate in fruit ripening (Di Matteo et al., 2003). The crunchiness of unripe Friariello meets the consumer taste and expectations who prefers it to the fully ripe fruit, in which the physiological process of cell walls softening has been completed (Schifferstein et al., 2019).

### Friariello Ripening Process: Flavor and Aromatic Bouquet Variation

The sweetness in vegetable products is mainly due to the synthesis and accumulation of large amount of carbohydrates during maturation (fructose, sucrose, glucose, galactose, maltose, and lactose) and, to a lesser extent, to a certain number of amino acids (alanine, valine, isoleucine, threonine, leucine, serine, arginine, asparagine, glutamine, lysine, cysteine, methionine, tryptophan, phenylalanine, glycine, and histidine). Carbohydrates and acids play a fundamental role in determining the accumulation of metabolites such as volatiles and pigments in ripening. Most carbohydrates are metabolized in the glycolysis and TCA cycle pathways during the early stages of development. Instead ribose, arabinose, raffinose and sucrose accumulate and starch is broken down in the late stages of ripening, as seen in previous researches (Martínez-Esteso et al., 2011; Li et al., 2019b). In this study, several proteins particularly up regulated in the final stage of maturation, belong to the functional group of TCA cycle. In particular, the enzyme that catalyzes the reversible conversion between oxaloacetate and malate in the TCA cycle, Malate dehydrogenase, shows a marked upregulation during the Friariello maturation, suggesting that ATP may be required as an energy source in this process (Aizat et al., 2013). Accordingly, enzymes such as Fructokinase-2, Putative fructokinase 6, chloroplastic Phosphotransferase, Phosphotransferase and Sucrose synthase are found only in fully mature Friariello. In contrast, the fructose-bisphosphate aldolase and the enolase have a constant decrease during chromoplast differentiation. In addition to carbohydrates and amino acids, there are other compounds capable of modulating the sweetness of plant species. Thaumatin, for example, is a protein 100,000 times sweeter than sucrose on a molar basis. This super-sweet protein is used in the food industry as a sweetener and flavor enhancer and in low-calorie diets (Firsov et al., 2016). The great abundance of this protein can definitely explain the particular sweetness and intense flavor of the G Friariello that is lost during the maturation when the abundance of Thaumatin suddenly decays (Figure 2E).

One of the greatest sources of compounds involved in food sensory properties is the protein catabolism, done by proteases to produce peptides that are then hydrolyzed into amino acids by peptidases. These not volatile compounds cannot be perceived by the olfactive receptors; however, they are perceived by the gustative nerve and constitute a very important factor in food palatability. Peptides can confer salty, umami, sour, sweet, or bitter flavors to fruits and vegetables. In addition, the amino acids breakdown leads directly to a large number of volatile compounds. Together with the organic acids, which are the primary contributors to the fruits sour taste, some amino acids like aspartic and glutamic acids can contribute to the sensation of sourness. Aspartate aminotransferase plays an important role in the synthesis or degradation of amino acids and can catalyze the reciprocal conversion between keto acids and amino acids (El Hadi et al., 2013). The exclusive presence of Aspartate aminotransferase in R Friariello, may indicate an accumulation of glutamate in the full riped fruit, with probable alteration of the flavor as seen in the literature (Zhang et al., 2013). Indeed, the up-regulation of aspartate aminotransferase could play an important role in amino acid accumulation in the fruit ripening process. As already confirmed in other non-climacteric fruits, such as the strawberry (Li et al., 2019a), a considerable amount of ribosomal subunits and elongation factors are expressed exclusively or up-regulated in the green stage of Friariello pepper, with the exception of the 40S-ribosomal protein S9 which is particularly abundant only in the stage of complete maturation. On the other hand, some proteins such as Peptidase A1 domain-containing protein, Serpin-ZX, Carboxypeptidase, and beta-hexosaminidase show an increase in expression in mature Friariello (Supplementary Table 1). Beta-hexosaminidase belong to a glycosidases class of enzymes that act on short chain oligosaccharides present in glycoproteins and are presumed to play a role in fruit softening during ripening (Jagadeesh et al., 2004). Proteasome is associated with the degradation of ubiquitinated proteins. In this work, several proteasome subunits are more expressed during the fruit veraison, plausibly due to the massive structural and functional conversion that occurs (Ling and Jarvis, 2015).

The metabolism of fatty acids is involved in the formation of volatile aromatics. Esters, alcohols, aldehydes, and terpenoids are mainly synthesized during the metabolism of fatty acids and amino acids (El Hadi et al., 2013). The proteins belonging to this functional group show an increasing expression trend during the ripening of the Friariello pepper, indicating an accumulation of volatile aromatic compounds and a marked alteration in the ripe fruit flavor. In agreement with the proteomics data, the results of metabolomics confirmed the more intense synthesis or lower degradation of lipids (Table 1). A complex mixture of volatile compounds is responsible for the fruit aroma, the expression of which is not only due to genetic and physiological factors internal to the plant. In fact, environmental factors and soil characteristics largely influence the organoleptic characteristics of the final product (Rodríguez-Burruezo et al., 2010). Some important aromas are produced as a result of a blend of several components in proper balance and not due to the presence of a unique compound (Dresow and Böhm, 2009). Volatile compounds detected by GC-MS analysis highlighted a substantial alteration in the aromatic bouquet of Friariello during maturation (Supplementary Table 3 and Figure 4B). In particular, the typical consumption phase, the green one has a greater quantity of butanol, 1 3 5-cycloheptatriene, dimethylheptane, α-pinene, furan-2-penthyl, ethylhexanol, 3-carene, Isobutyl-3-methoxy-pyrazine, decane 2,3,5,8-tetramethyl, 2,4-difluorobenzoic acid 2 ethyl hexyl ester. As can be seen in Figure 4B, it is the combination and relative abundances of the various compounds that determine the peculiar aroma and flavor of green Friariello, particularly appreciated in the regions of southern Italy. The aromatic bouquet of Friariello pepper undergoes significant changes during maturation. The concentrations of volatile compounds (Supplementary Table 3 and Figure 4B) show some significant differences. In fact its composition is linked to the hints of green, typically associated with the sensations of fresh and pleasant on the palate, decreased or disappeared during ripening while the fruity and sweet aroma was higher during the veraison phase and then disappeared in the mature Friariello. The data are in agreement with previous studies (Mazida et al., 2005), and confirm the peculiar characteristics of Friariello verse justifying its preferential consumption in the early stage of ripening.

## Conclusion

The quality of the flavor of fruit and vegetables depends more than just on smell or taste but on a combination of several factors. The taste is determined by the relative content of sugars, different amino acids and nucleotides, acids, salts and bitter compounds that combine with each other and that are decidedly altered during maturation. Although usually the ripening process makes the fruits more palatable for human consumption, in some cases, such as the Friariello pepper, it is preferred to consume it at an early stage. In this work, for the first time, the proteomic and metabolomic analysis of Friariello during the maturation process allowed to highlight the physiological alterations. In particular, the super sweet protein Thaumatin and a series of VOCs, abundant in the green Friariello, can explain the particular sweet and fresh flavor of this pepper. In fact, in the green stage the proteomic and metabolomic analyses have highlighted the organoleptic and nutritional characteristics peculiar of this fruit distinctive of a specific agro-cultural territory.

The authors declare that the research was conducted in the absence of any commercial or financial relationships that could be construed as a potential conflict of interest.

## Data Availability Statement

The datasets presented in this study can be found in online repositories. The names of the repository/repositories and accession number(s) can be found below: ProteomeXchange, PXD025181.

## Author Contributions

All authors make substantial contributions to conception and design of the manuscript, jointly interpreted the data, contributed to the manuscript critical evaluation, revision, read, and approved the submitted version. MT, RS, and CG conceptualized and designed the study. AA, AI, and GP conducted the samples analyses. DZ and JJ-N performed the statistical data analysis. MT wrote the first draft of the manuscript.

## Conflict of Interest

The authors declare that the research was conducted in the absence of any commercial or financial relationships that could be construed as a potential conflict of interest.

## Footnotes

1 www.matrixscience.com

2 https://www.R-project.org/

3 https://mapman.gabipd.org/app/mercator

## Supplementary Material

Supplementary Figure 1 Matrix-assisted laser desorption/ionization spectra of hexane extracts of green **(A)**, green/red **(B)**, and red **(C)** pepper.

Supplementary Figure 2 TIC chromatograms by GC-MS analysis of hexane extracts from green pepper, mixed (green/red) and red peppers. An overlap of TIC chromatograms is reported.

Supplementary Figure 3 TIC chromatograms by GC-MS analysis of volatile compounds extracted by SPME fiber from green, green/red, and red peppers. An overlap of TIC chromatograms is reported.

Supplementary Table 1 Table with list of Uniprot ID, protein name, Average Normalized Empai%, Standard Deviation and CV% for the 668 proteins identified in the three stages of maturation of Friariello analyzed (G–green, G/R-green/red, and R-red).

Supplementary Table 2 List of semi-volatile compounds detected by GC-MS analysis. The retention time, analyte identification is listed whereas TIC peak area is reported for each sample, i. e. green, green/red, and red Friariello peppers.

Supplementary Table 3 List of volatile compounds detected by GC-MS analysis. The retention time, analyte identification is listed whereas TIC peak area is reported for each sample, i. e. green, green/red, and red peppers. For each molecule is reported the specific aroma whereas it was found.
