# Supplementary figures and images for: Why Consumers Prefer Green Friariello Pepper: Changes in the Protein and Metabolite Profiles Along the Ripening
Source: Front Plant Sci. 2021 Apr 30;12:668562. doi: 10.3389/fpls.2021.668562 (PMC8121147; doi:10.3389/fpls.2021.668562)

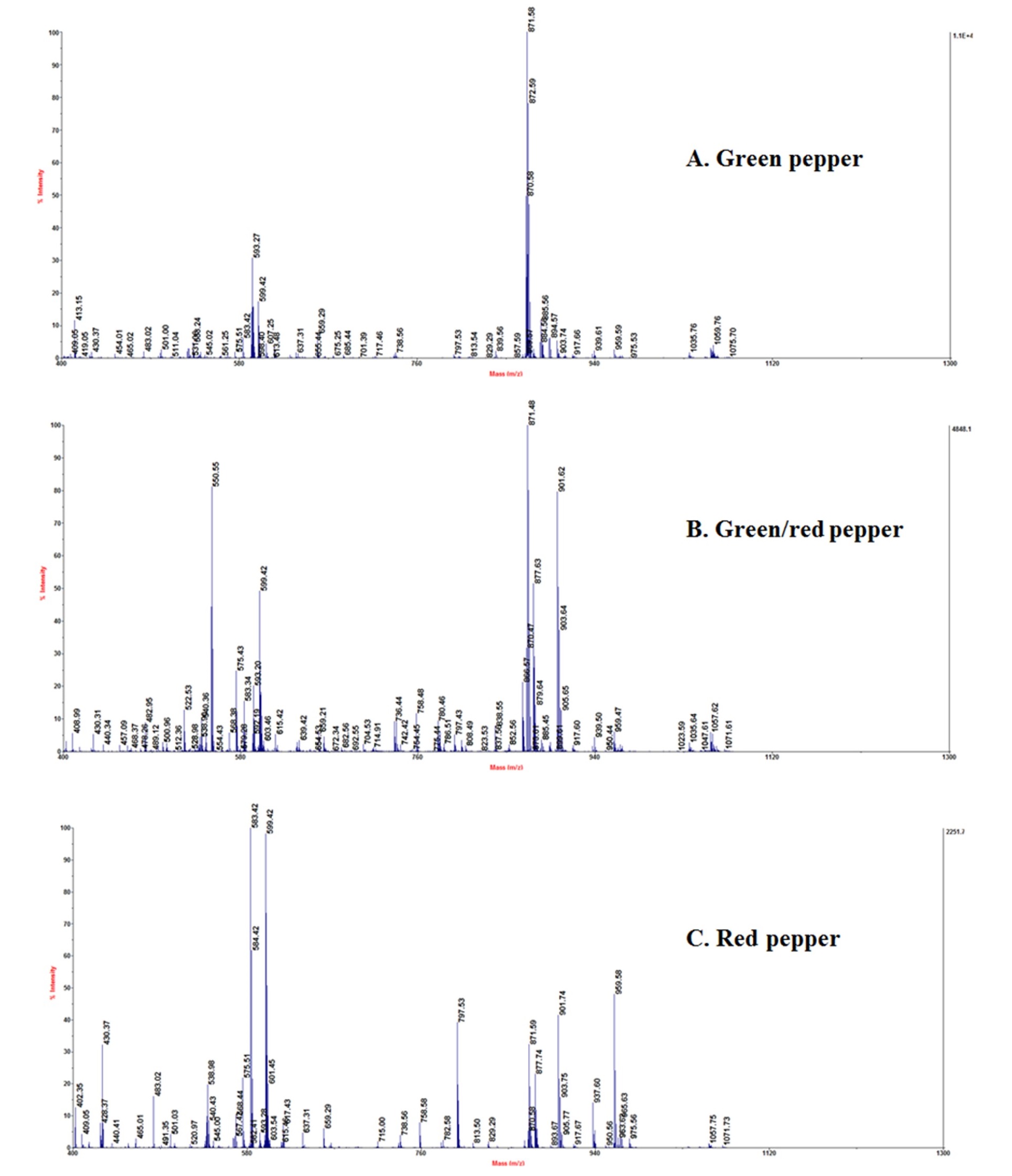

Supplement: Supplementary Figure 1 — Matrix-assisted laser desorption/ionization spectra of hexane extracts of green (A), green/red (B), and red (C) pepper. [file Image_1.JPEG]

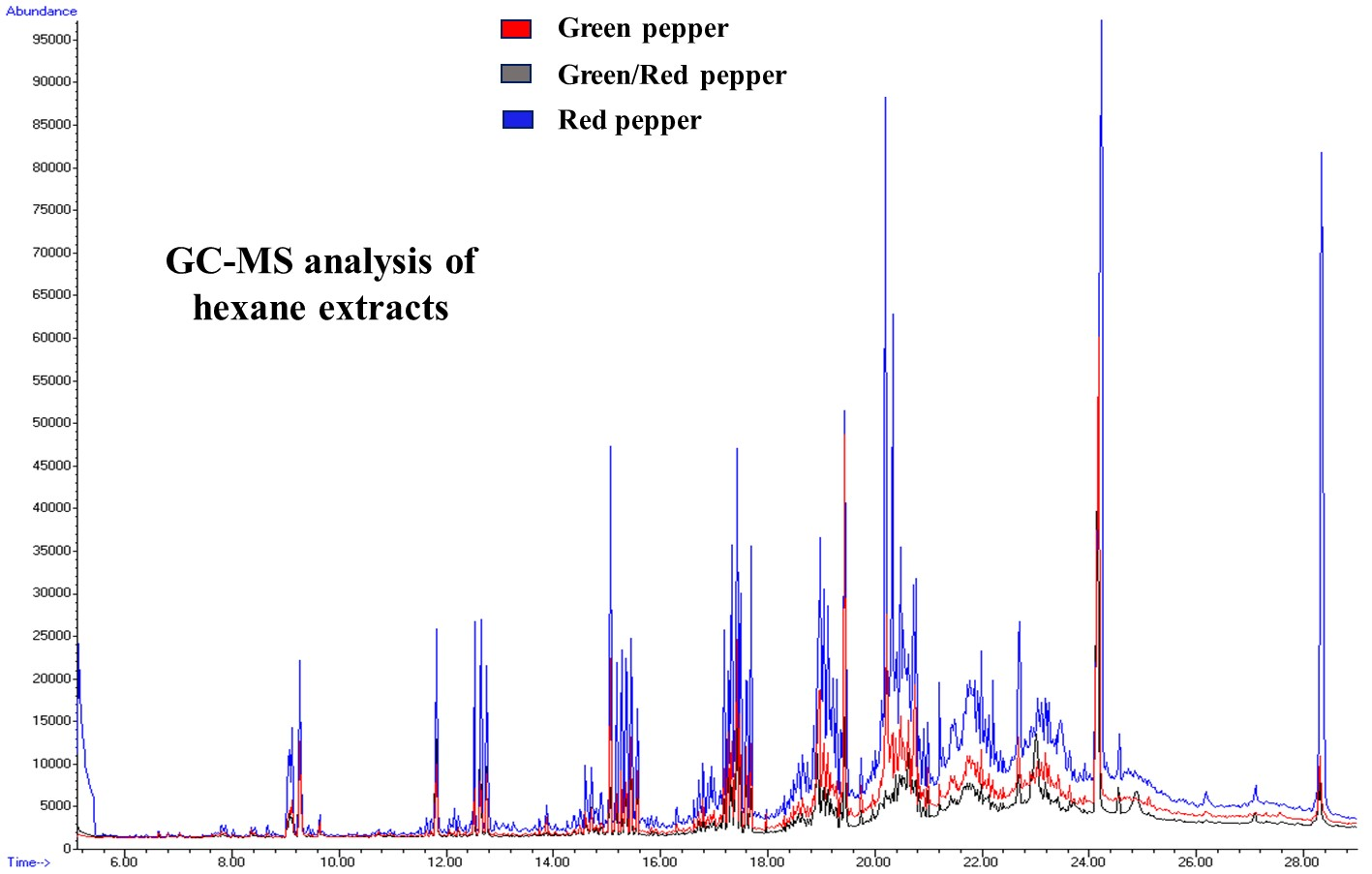

Supplement: Supplementary Figure 2 — TIC chromatograms by GC-MS analysis of hexane extracts from green pepper, mixed (green/red) and red peppers. An overlap of TIC chromatograms is reported. [file Image_2.TIF]

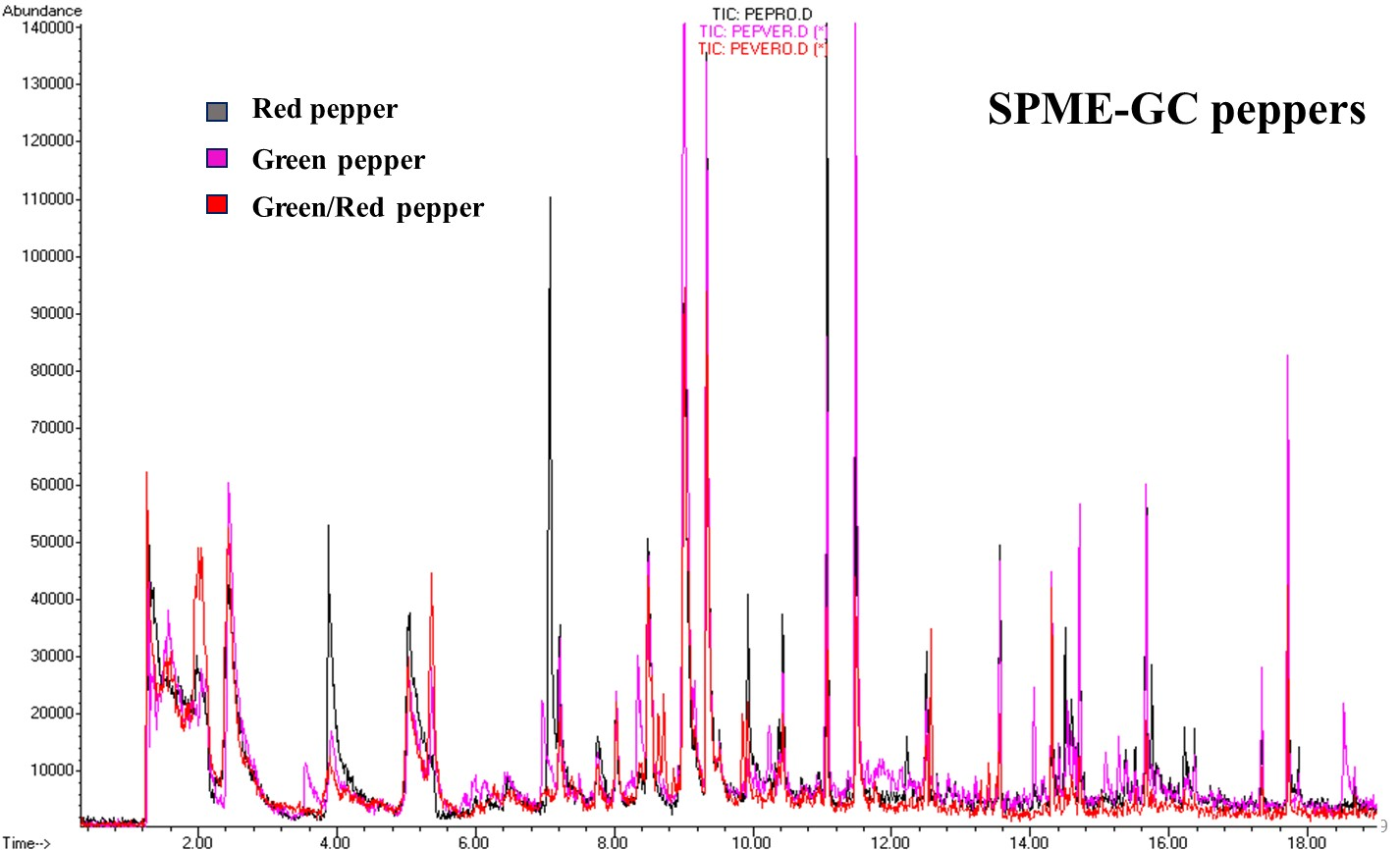

Supplement: Supplementary Figure 3 — TIC chromatograms by GC-MS analysis of volatile compounds extracted by SPME fiber from green, green/red, and red peppers. An overlap of TIC chromatograms is reported. [file Image_3.TIF]
