# Supplementary material for: Why Consumers Prefer Green Friariello Pepper: Changes in the Protein and Metabolite Profiles Along the Ripening
Source: Front Plant Sci. 2021 Apr 30;12:668562. doi: 10.3389/fpls.2021.668562 (PMC8121147; doi:10.3389/fpls.2021.668562)
Supplement: Supplementary Table 2 — List of semi-volatile compounds detected by GC-MS analysis. The retention time, analyte identification is listed whereas TIC peak area is reported for each sample, i. e. green, green/red, and red Friariello peppers. [file Table_2.DOCX]

|  | **Analyte** | **Green** | **Green/ Red** | **Red** |
| --- | --- | --- | --- | --- |
| **Alkanes and alkenes components** | | | | |
| **7,8** | Decane, 4-methyl- | 9,04E+05 |  | 9,71E+05 |
| **7,9** | Octane, 3,5-dimethyl- | 1,26E+06 |  | 2,37E+06 |
| **8,03** | Hexane, 3-ethyl- | 2,40E+05 |  |  |
| **8,04** | 4-Decene, 7-methyl-, (E)- |  |  | 4,45E+05 |
| **8,56** | Decane, 2,9-dimethyl- | 1,25E+05 |  |  |
| **8,66** | Decane | 8,55E+05 |  | 2,54E+06 |
| **8,77** | Decane, 2,4,6-trimethyl- | 2,63E+05 |  | 1,05E+06 |
| **9,02** | 1-Octene, 3,7-dimethyl- | 1,30E+06 |  | 2,87E+06 |
| **9,42** | Undecane | 5,58E+05 |  | 1,80E+06 |
| **9,5** | Octane, 3,5-dimethyl-/Undecane | 2,12E+05 |  | 6,24E+05 |
| **9,6** | Undecane, 4-methyl- |  |  | 1,12E+06 |
| **10,96** | Dodecane | 3,77E+05 |  | 6,26E+05 |
| **11,17** | Tridecane, 4-methyl- | 1,63E+05 |  |  |
| **11,51** | Decane, 2,3,5,8-tetramethyl- | 3,05E+05 |  | 6,73E+05 |
| **11,8** | Benzene, 1,3-bis(1,1-dimethylethyl)- | 1,71E+06 | 3,33E+06 | 3,98E+06 |
| **12** | Undecane, 2-methyl- |  |  | 2,69E+06 |
| **12,14** | Dodecane, 2,6,11-trimethyl-/ | 4,04E+05 |  | 7,98E+05 |
| **12,64** | 3-Octadecene, (E)- | 2,02E+06 |  |  |
| **12,74** | 1-Nonene, 4,6,8-trimethyl- |  | 1,84E+06 |  |
| **12,9** | Dodecane, 2,6,11-trimethyl- |  |  | 1,62E+06 |
| **13,75** | Hexadecane |  | 4,21E+05 |  |
| **14,5** | Undecane, 2,3,5,8-tetramethyl- | 7,55E+05 | 1,24E+06 |  |
| **15,9** | Heptadecane, 2,6-dimethyl- | 3,84E+05 |  | 7,82E+07 |
| **17,41** | 1-Docosene | 4,53E+06 |  | 1,31E+06 |
| **20,76** | 2-Methyl-Z-4-tetradecene | 6,00E+06 | 5,03E+06 |  |
| **Alcohols** | | | | |
| **6,8** | 1,2,6-Hexanetriol | 1,35E+06 | 1,20E+06 | 2,18E+06 |
| **9,23** | 1-heptanol, 2-butyl- |  |  | 1,30E+06 |
| **9,62** | Cyclohexanol, 5-methyl-2-(1-methylethyl)-, [1S-(1a,2a,5ß)]- | 4,22E+05 |  |  |
| **12,34** | 2-Hexen-1-ol, 2-ethyl- | 2,69E+05 |  |  |
| **12,5** | 2-Pentyl-1-octanol |  |  | 5,75E+06 |
| **12,51** | 1-Octanol, 2-butyl- | 1,51E+06 | 1,71E+06 | 6,77E+06 |
| **12,64** | 2-Isopropyl-5-methyl-1-heptanol | 1,43E+05 | 2,40E+06 | 8,52E+06 |
| **12,76** | 11-Methyldodecanol | 2,02E+06 |  |  |
| **15,04** | Phenol, 2,4-bis(1,1-dimethylethyl)- | 3,94E+06 | 1,73E+06 | 1,13E+07 |
| **15,17** | 2-Hexyl-1-octanol | 1,21E+06 | 1,07E+06 |  |
| **15,46** | 3-Hexyl-1-octanol | 2,01E+06 |  |  |
| **17,18** | 1-Decanol, 2-hexyl- | 1,63E+06 | 2,23E+06 |  |
| **17,6** | 2-Hexyl-1-decanol | 1,83E+06 | 1,40E+06 |  |
| **19,1** | 11-Methyldodecanol |  | 3,46E+06 |  |
| **19,44** | Falcarinol | 7,38E+06 |  | 1,21E+07 |
| **22,6** | Pyrazol-5-ol, 1-acetyl-3,4-dimethyl-, acetate (ester) |  |  | 1,26E+07 |
| **24,57** | 12-Methyl-E,E-2,13-octadecadien-1-ol |  | 1,48E+06 |  |
| **24,57** | 12-Methyl-E,E-2,13-octadecadien-1-ol |  | 1,48E+06 |  |
| **Alogenate and sulphate compounds** | | | | |
| **14,81** | Sulfurous acid, hexyl pentadecyl ester | 5,38E+05 |  |  |
| **14,69** | 1-Iodo-2-methylundecane | 1,27E+06 |  |  |
| **15,34** | 1-Iodo-3-methylundecane | 2,34E+06 |  |  |
| **9,1** | 2-Undecanethiol, 2-methyl |  | 6,23E+05 |  |
| **19,42** | Phenol, 2-benzyloxy-3,6-difluoro- |  | 2,81E+06 |  |
| **9,04** | 2,6-Dimethyl-6-trifluoroacetoxyoctane | 9,22E+05 |  | 3,25E+06 |
| **Amine and amide compounds** | | | | |
| **22,66** | 9-Octadecenamide, (Z)- | 4,36E+06 | 3,75E+06 |  |
| **24,19** | 13-Docosenamide, (Z)- | 2,38E+07 | 2,21E+07 | 6,71E+07 |
| **7,09** | Hydroxylamine, O-(3-methylbutyl)- | 3,91E+05 |  |  |
| **20,23** | 1-Octadecanamine | 2,50E+07 |  |  |
| **Ketons** | | | | |
| **6,63** | Ethanone, 1-(3-ethyloxiranyl)- | 1,25E+06 | 1,13E+06 | 1,61E+06 |
| **8,37** | Ketone, isopropylidenecyclopropyl methyl | 2,95E+05 |  |  |
| **9,25** | 1-(2,4-Dimethyl-furan-3-yl)-ethanone | 6,78E+05 | 2,68E+05 | 7,34E+05 |
| **Heterocycles** | | | | |
| **7,03** | Furan, tetrahydro-2,2,4,4-tetramethyl- | 5,29E+05 | 4,61E+05 |  |
| **18,9** | Thiophene, 2,3-dimethyl- |  |  | 4,13E+07 |
| **28,3** | 1,6-Dimethyl-5-oxo-1,2,3,5-tetrahydroimidazo[1,2-a]pyrimidine | 3,08E+06 | 2,82E+06 |  |
| **Acids** | | | | |
| **18,69** | n-Hexadecanoic acid | 3,11E+06 | 7,84E+06 | 1,60E+07 |
| **20,32** | Octadecanoic acid |  |  | 1,79E+07 |
| **20,74** | Oleic Acid |  | 2,16E+06 |  |
| **Sterols** | | | | |
| **23** | ß-Sitosterol |  | 9,17E+06 |  |
| **Vitamins** | | | | |
| **28,33** | Vitamin E |  | 2,07E+06 | 8,91E+06 |

**Supplementary Table 2:** List of semi-volatile compounds detected by GC-MS analysis. The retention time, analyte identification is listed whereas TIC peak area is reported for each sample, i. e. green, green/red and red Friariello peppers.
