# Supplementary material for: Why Consumers Prefer Green Friariello Pepper: Changes in the Protein and Metabolite Profiles Along the Ripening
Source: Front Plant Sci. 2021 Apr 30;12:668562. doi: 10.3389/fpls.2021.668562 (PMC8121147; doi:10.3389/fpls.2021.668562)
Supplement: Supplementary Table 3 — List of volatile compounds detected by GC-MS analysis. The retention time, analyte identification is listed whereas TIC peak area is reported for each sample, i. e. green, green/red, and red peppers. For each molecule is reported the specific aroma whereas it was found. [file Table_3.DOCX]

|  |  | **Green** | **Green/Red** | **Red** |  |
| --- | --- | --- | --- | --- | --- |
| **ID** | **Time (min)** | **Peak area** | **Peak area** | **Peak area** | **Aroma** |
| bis-dicloro-methyl-ester | 1.99 |  | 1.42E+06 |  | fruity |
| butanol | 2.45 | 3.52E+06 | 3.17E+06 | 2.31E+06 | green notes/medicinal |
| hydroxylamine-o-isopenthyl | 3.85 |  | 7.21E+05 |  | - |
| 1 3 5-cycloheptatriene | 3.92 | 4.39E+05 |  |  | - |
| dimethyleptene | 5.36 | 5.25E+05 | 1.66E+06 |  | pungent |
| α-pinene | 7.21 | 8.30E+05 | 4.74E+05 | 6.78E+05 | pine wood |
| β-pinene | 8 | 5.57E+05 | 5.75E+05 | 4.49E+05 | pine wood |
| furan-2-penthyl | 8.34 | 8.39E+05 |  |  | green |
| eptan2-4-dimethyl | 8.64 |  | 1.26E+05 |  | - |
| decane 4-methyl | 8.71 |  | 3.84E+05 |  | candle like.citrus like |
| hetyl-hexanol | 9 | 7.25E+06 | 4.49E+06 | 3.86E+06 | green |
| β-ocimene | 9.33 | 3.40E+06 | 2.56E+06 | 3.45E+06 | rancid |
| 3-carene | 9.51 | 5.15E+04 | 1.76E+04 | 1.11E+05 | ruberry |
| 5-tridecane | 9.85 |  | 3.10E+05 |  | - |
| butil-octanol | 9.92 |  | 2.80E+05 |  | pungent |
| Isobutil-3-methoxy-pirazine | 11.47 | 3.36E+06 | 1.26E+06 | 2.14E+06 | green bell pepper |
| 1.3 tert-buthyl-benzene | 12.57 |  | 4.93E+05 |  | Sweet |
| 1-octanol-2-esil | 13.52 |  | 4.10E+05 |  | pungent |
| decane 2.3.5.8-tetramethyl | 14.05 | 3.12E+05 |  |  | candle like.citrus like |
| α-capaene | 14.31 | 5.95E+05 | 4.11E+05 | 6.72E+05 | woody |
| tau-elemene | 14.5 |  |  | 2.74E+05 | floral |
| 3-hidroxy-1-propenil-ciclopentanone | 14.71 | 6.55E+05 | 1.69E+05 | 6.82E+05 | spicy |
| spiro[5-5]undec2-ene3-7-7-trimetil1-1-metilene | 15.75 |  |  | 2.92E+05 | - |
| butil-5-hexyl-octa-idro-hyndene | 17.72 | 1.08E+06 | 5.06E+05 |  | - |
| 2.4-difluoro benzoic acid 2 ethyl hexyl ester | 18.51 | 3.24E+05 |  |  | - |

**Supplementary Table 3:** List of volatile compounds detected by GC-MS analysis, the retention time, analyte identification is listed whereas TIC peak area is reported for each sample. i. e. green, green/red and red peppers. For each molecule is reported the specific aroma whereas it was found.
